# Exploring the Correlation Between Splenomegaly and Lung Involvement in COVID-19: A Retrospective Study

**DOI:** 10.7759/cureus.55415

**Published:** 2024-03-02

**Authors:** Bharathi Priya Raju, Balaji Selvaraj, Sharmila Murugesan, Suhasini Balasubramaniam, Sowmiya PK, Pravin Kumar Raviganesh, Rajasekaran Sivaprakasam, Sangeetha Balaji, Rupert Nithin Fernando, Swaminathan Ramasubramanian

**Affiliations:** 1 Radiodiagnosis, Government Stanley Medical College and Hospital, Chennai, IND; 2 Radiodiagnosis, Government Medical College, Omandurar Government Estate, Chennai, IND; 3 Internal Medicine, Government Medical College, Omandurar Government Estate, Chennai, IND; 4 Radiodiagnosis, Jawaharlal Institute of Postgraduate Medical Education and Research, Puducherry, IND; 5 Pharmacology, Government Medical College, Omandurar Government Estate, Chennai, IND; 6 Radiodiagnosis, Davao Medical School Foundation, Davao, PHL

**Keywords:** spleen size, radiology, retrospective study, ct imaging, lung involvement, splenomegaly, covid-19

## Abstract

Background

Coronavirus disease 2019 (COVID-19), resulting from the severe acute respiratory syndrome corona virus 2 (SARS-CoV-2), has not only shown substantial effects on the respiratory system but also on extrapulmonary systems, including cardiovascular, gastrointestinal, hematological, and immune responses, notably spleen enlargement. The connection between the enlargement of the spleen and pulmonary complications in individuals with COVID-19 is still not well elucidated, with current studies offering divergent conclusions.

Objective

This study aims to elucidate the correlation between splenomegaly, as assessed by computed tomography (CT) imaging, and the extent of lung involvement (LI) in COVID-19 patients, thereby offering insights into potential prognostic indicators.

Methodology

A hospital-based, cross-sectional, retrospective study was conducted involving 1058 symptomatic COVID-19 patients confirmed by reverse transcriptase-polymerase chain reaction (RT-PCR), aged 18 years and above. CT imaging was utilized to evaluate spleen size and LI. Statistical analyses, including Pearson correlation and simple linear regression, were performed to explore the relationship between spleen size and LI.

Results

The study cohort exhibited a mean spleen size of 9.49 cm and a mean LI score of 0.272. The Pearson correlation coefficient was calculated at 0.0495, indicating a marginal positive correlation between spleen size and LI. Regression analysis demonstrated a minimal impact of spleen size on LI, with spleen size accounting for only 0.2% of the variance in LI scores.

Conclusions

The study found a slight, statistically non-significant correlation between splenomegaly and LI in COVID-19 patients, suggesting that while splenic enlargement may reflect systemic disease involvement, it is not a strong independent predictor of lung damage extent. The findings highlight the complexity of extrapulmonary manifestations and highlight the need for additional research to fully understand the implications of splenic involvement in COVID-19.

## Introduction

The spleen, an integral component of the human lymphatic system, plays a multifaceted role in maintaining homeostasis. The functions of the spleen span from the synthesis and storage of red blood cells to the elimination of aberrant cells from circulation, actively participating in immune responses through antibody generation and pathogen filtration [1]. Various factors, including age, general health status, and specific medical conditions, influence the size and appearance of the spleen. In the realm of radiology, essential diagnostic tools such as computed tomography (CT) scans, ultrasonography (USG), and magnetic resonance imaging (MRI) contribute significantly to the identification and characterization of splenic abnormalities, particularly splenomegaly [1]. CT scans, widely employed for their capacity to assess spleen morphology and size, are instrumental in identifying potential causes of splenomegaly [1]. Notably, ultrasonographic findings denote splenomegaly when the coronal plane diameter exceeds 12 cm [2].

Splenomegaly, defined as the abnormal enlargement of the spleen, can be attributed to a myriad of causes, including infectious agents such as bacterial endocarditis, infectious mononucleosis, HIV, hepatitis C, malaria, tuberculosis, and histiocytosis [3,4]. Unusual manifestations of splenomegaly, such as in dengue fever or as an indicator of portal hypertension and advanced chronic liver disease in HIV patients, demonstrate the diverse range of the presentations of this condition [5,6].

The coronavirus disease 2019 (COVID-19) pandemic, caused by the severe acute respiratory syndrome corona virus 2 (SARS-CoV-2), has not only inflicted substantial morbidity and mortality but has also revealed extrapulmonary manifestations, including splenomegaly [7]. While COVID-19 primarily affects the respiratory system, the involvement of other organs and systems, such as the cardiovascular, gastrointestinal, hematological, and immune systems, has been documented [8]. The spleen, being a vital organ of the immune system, can exhibit enlargement in response to systemic inflammation or cytokine storms associated with severe COVID-19 cases [7,8].

The relationship between splenomegaly and pulmonary manifestations in COVID-19 patients is yet to be fully elucidated, as current studies present divergent results. Specifically, Samir et al. concluded that splenomegaly does not significantly correlate with COVID-19 [7] in contrast to Aksu et al. who reported a frequent association between splenomegaly and COVID-19 [8]. Consequently, this study aims to elucidate the relationship between splenomegaly and lung involvement (LI) in COVID-19 patients through a retrospective analysis of CT scans and clinical data. We hypothesize that splenomegaly may serve as an indicator of the extent of lung damage and inflammation, providing valuable prognostic implications.

## Materials and methods

Study setting and design

This retrospective cross-sectional study was conducted within a hospital setting, primarily focusing on the analysis of CT chest images. Approval from the Institutional Ethics Committee was obtained, with reference to IEC No. 108/IEC/GOMC/2023. The research strictly adhered to the ethical guidelines established by the institutional research committee and conformed to the principles outlined in the 1964 Helsinki Declaration, including any subsequent amendments or equivalent ethical standards. The investigation took place at the Department of Radiodiagnosis, situated within the Government Medical College at Omandurar Government Estate, Chennai. This institution is affiliated with The Tamil Nadu Dr. M.G.R. Medical University and served as a designated COVID-19 Care Center at the onset of the pandemic.

Study period

The study was carried out from April 2021 to June 2021, capturing a specific timeframe for comprehensive data analysis.

Participants

The participant pool included adults aged 18 years and above.

Inclusion criteria

Patients eligible for inclusion in this investigation had to be 18 years of age or older upon admission to the healthcare facility. Furthermore, candidates were required to exhibit clinical symptoms consistent with COVID-19, including fever or chills, cough, shortness of breath or difficulty breathing, fatigue, muscle or body aches, headache, new loss of taste or smell, sore throat, congestion or runny nose, nausea or vomiting, and diarrhea [9]. An essential criterion for inclusion was a confirmed positive test result for COVID-19.

Exclusion criteria

Individuals below 18 years of age, those not displaying clinical symptoms consistent with COVID-19, or individuals lacking a confirmed positive RT-PCR test for COVID-19 were excluded from the study [9].

Sample size

A total of 1058 patients were selected from the eligible cohort that met the specified criteria using convenience sampling.

CT chest imaging protocol

A non-contrast-enhanced chest CT scan was executed with patients in the supine position, utilizing a 16-section multidetector CT scanner (Aquilion Lightning model TSX-035A, Toshiba America Medical Systems, Tustin, USA). Patients maintained a single inspiratory breath-hold during the procedure. Image analysis employed Vitrea software version 6.5.99 (Vital Images, Inc., Otawara, Japan), with specific parameters: a window width ranging from 1000 to 2000 Hounsfield units (HU) and a window level adjusted from -700 to -500 HU. The examination covered the entire lung region, from the apex to the costophrenic angles. Initial interpretation involved two radiologists with a combined 15 years of experience, followed by collaborative reassessment with residents and interns. A double-blinded method facilitated image distribution for review. Interpretative disagreements were resolved through adjudication by a senior radiologist with 20 years of experience.

Quantification of pulmonary involvement and splenomegaly

LI was assessed in 20 sections, with scores based on opacification: two for over half involvement and one for less than half. The total maximum score was 40, each unit representing 2.5% of affected lung parenchyma [10]. The longest coronal diameter, observed in axial images of the CT chest, determined the measurement. The spleen, encompassed in all CT chest series planning, was considered enlarged if its coronal oblique length exceeded 12 cm [2].

Data management

Information from CT images and patient records was systematically entered into a Microsoft Excel spreadsheet (Microsoft® Corp., Redmond, USA), following stringent confidentiality and privacy protocols.

Statistical analysis

The statistical analysis was conducted using Python 3.9 (Python Software Foundation, Wilmington, USA) and incorporated several libraries, including *pandas* for data manipulation, *numpy* for numerical computations, *scipy* for statistical tests, and *matplotlib* for data visualization. Initially, descriptive statistics were generated to summarize the dataset characteristics. For spleen size and LI, measures such as mean, standard deviation, minimum, 25^th^ percentile, median, 75^th^ percentile, and maximum were calculated to provide a detailed understanding of the data distribution. To visually assess the relationship between spleen size and LI, a scatter plot was created. To quantify this relationship, the Pearson correlation coefficient (r) was calculated. Further, a simple linear regression analysis was employed to model the relationship between spleen size (predictor) and LI (outcome). Additionally, we examined LI in patients with splenomegaly, with descriptive statistics. The Shapiro-Wilk test was utilized to assess the normality of the LI distribution among these patients.

Ethical considerations

The study adhered to our institution's ethical guidelines, prioritizing the confidentiality and anonymity of patient data. For anonymity, patient identification details were eliminated using the Picture Archiving and Communications System (PACS) software (GE Healthcare, Chicago, USA). Subsequently, unique random numbers were assigned to each image and clinical data record to facilitate linking while preserving anonymity. The data was securely stored in the Department of Radiodiagnosis computer system within a password-protected folder. All procedures involving human participants were conducted in accordance with the ethical standards set by the institutional and/or national research committee.

## Results

In this study, we investigated the potential association between spleen size, as measured via CT scans, and the degree of LI in COVID-19 patients.

The analysis was predicated on a meticulously collated dataset comprising measurements derived from 1058 patients. Table 1 presents a comprehensive overview of our findings, with the spleen size of the cohort averaging at 9.49 cm (standard deviation (SD) = 1.64 cm, range = 5.00-14.60 cm). In parallel, LI was quantified on a scale from 0 to 1, with a mean value of 0.272 (SD = 0.246), indicating the relative extent of pulmonary affliction due to COVID-19.

**Table 1:** Descriptive statistics for spleen size and lung involvement

Variable	Count	Mean	Standard Deviation	Min	25^th ^Percentile	Median	75^th^ Percentile	Max
Spleen size	1058	9.49	1.64	5.00	8.50	9.50	10.50	14.60
Lung involvement (LI)	1058	0.272	0.246	0.00	0.05	0.225	0.450	1.00

**Figure 1:**
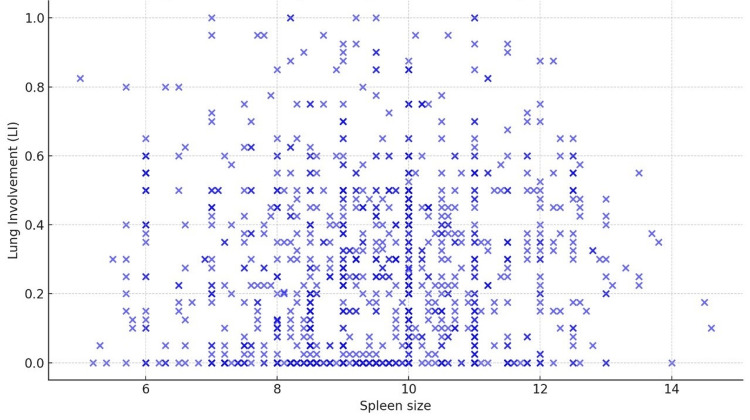
Scatter plot demonstrating the relationship between spleen size and lung involvement in COVID-19 patients

A visual inspection of the association between spleen size and LI was facilitated through a scatter plot, as denoted in Figure 1. This graphical representation suggested a marginal positive correlation between the two variables, albeit accompanied by significant variability across the dataset. The nuances of this relationship were further explored through rigorous statistical analyses.

Correlation and regression analysis

The Pearson correlation coefficient (r) was computed to be 0.0495, signifying a marginal linear association between spleen size and LI. To further delineate this relationship, a simple linear regression analysis was conducted, positing spleen size as the predictor variable for LI. The derived model equation,

$$LI = 0.2014 + 0.0074 &times; Spleen size$$

underscores the incremental impact of spleen size on LI. Table 2 elucidates the regression output, indicating an intercept of 0.2014 (p < 0.001) and a coefficient of 0.0074 for spleen size (p = 0.108). Although the coefficient for spleen size is positive, suggesting an increase in LI with larger spleen size, this association did not reach statistical significance at the conventional alpha level of 0.05. The model's R^2^ value of 0.002 intimates that spleen size accounts for a mere 0.2% of the variance in LI, highlighting the complexity of the pathophysiological interactions at play and the potential influence of other covariates not accounted for in the current model.

**Table 2:** Regression coefficients for the model

Variable	Coefficient	Standard Error	p-value
Intercept	0.2014	0.044	<0.001
Spleen size	0.0074	0.005	0.108

Analysis of LI in splenomegaly cases

An ancillary analysis was undertaken to scrutinize the characteristics of LI among patients manifesting with splenomegaly. Table 3 encapsulates the distributional properties of LI within this subset, indicating a mean LI of 0.3184 with a standard deviation of 0.2061. Table 3 affords a granular insight into the pulmonary impact of COVID-19 in patients with enlarged spleens, showcasing a range of LI from minimal to substantial.

**Table 3:** Distributional attributes of lung involvement in splenomegaly cases

Statistic	Value
Count	64.0000
Mean	0.3184
Std Dev	0.2061
Min	0.0000
25%	0.1500
Median	0.3250
75%	0.4750
Max	0.8750

Distribution normality test

**Figure 2:**
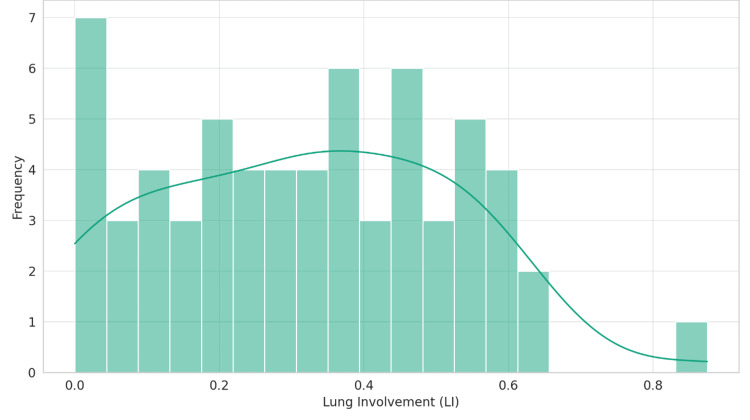
Distribution of lung involvement in cases of splenomegaly

To assess the distributional normality of LI amongst patients with splenomegaly, the Shapiro-Wilk test was employed, yielding a statistic of 0.9645 and a p-value of 0.0626. This outcome, while not definitively contravening the normality assumption, approaches the threshold of statistical significance, thereby advising caution in the interpretation of these results. Figure 2 graphically illustrates the distribution of LI in cases of splenomegaly, providing a visual complement to the statistical analysis.

## Discussion

The implications of COVID-19 on the spleen have emerged as a pivotal area of interest in recent research. Early observations from clinical settings indicated that COVID-19 might be associated with splenic abnormalities, presenting potential insights into both disease progression and the physiological response of the body [7,8]. In our study, in patients with splenomegaly, a condition often associated with more severe systemic manifestations in various diseases, the LI appeared slightly higher with a mean of 0.3184, compared to the overall cohort's mean of 0.272. However, this difference, while suggestive, is not pronounced enough to assert a direct clinical relevance without further study. Moreover, our normality test for the distribution of LI in splenomegaly cases returned a Shapiro-Wilk statistic close to the significance threshold, advising a degree of caution when drawing conclusions based on this distribution.

Batur et al.'s comprehensive investigation into the spleen's susceptibility to COVID-19 provided compelling findings. Subsequent CT scans demonstrated notable reductions in spleen size in their patient cohort. However, the consistency of HU values was maintained. The radiomics analysis from the study was even more enlightening, revealing discernible discrepancies in various splenic features, hinting at alterations in the parenchymal microstructure of the spleen [11]. This evidence brings forth the contention that the spleen could be systematically impacted by COVID-19, thus presenting it as a potential target organ. The swift transformation in spleen size and its microstructural facets elevates the importance of further probing into the long-lasting implications of the virus on splenic health [11]. Aksu et al. further reinforced the argument of splenic involvement in COVID-19, linking splenomegaly in patients with COVID-19-induced pneumonia to an increase in lung abnormalities. This suggests a heightened severity of LI, particularly evident in specific lung segments [8].

This connection between spleen and LI is not without complexities, though Xie et al. painted a different picture, with their comparative research indicating a decrease in spleen size, in terms of both length and thickness, in patients afflicted with the virus [12]. Contrasting this, Tahtabasi et al. identified an enlargement in splenic volume during the initial stages of COVID-19 infection, thereby emphasizing a direct relationship between splenic growth and infection severity, as corroborated by chest CT scans [13].

Previous research has emphasized the clinical significance of splenomegaly in the context of COVID-19 [11,14]. Conditions such as hemophagocytic lympho-histiocytosis (HLH) and its concomitant cytokine storms manifest alongside splenomegaly and pose severe implications for COVID-19 patients [15-17]. It is important to consider cases of pancytopenia along with splenomegaly observed on CT scans in COVID-19 patients. In such clinical presentations, the differential diagnosis may lean towards hairy cell leukemia (HCL) [14]. Misdiagnosis in these situations can have dire consequences, especially in the realm of a condition as multifaceted as COVID-19. The potential for splenomegaly to be a marker for HLH in emergency medical settings further underscores the gravity of the situation [14]. The manifestation overlap between HLH syndromes and severe COVID-19 symptoms, particularly in the context of inflammatory dysregulation and cytokine storms, is deserving of careful attention and study. This convergence may provide pathways to explore potential therapeutic strategies, in addition to furnishing a deeper understanding of disease progression [15-17].

In the broader context of research on COVID-19 and spleen interactions, our findings present a nuanced picture. While there appears to be a connection between spleen size and LI, the strength of this relationship, as evidenced by our dataset, is relatively weak. It is essential to juxtapose this with the studies mentioned earlier, which indicated systemic effects of COVID-19 on the spleen, leading to both size reductions and microstructural changes over time [11-13]. One might wonder whether the nuanced changes in spleen size, as observed in our study, might manifest more pronouncedly in the progression of the disease or other related sequelae.

The strengths of this study lie in its comprehensive approach to investigating the relationship between spleen size and LI in COVID-19 patients. It benefits from a large sample size of 1058 patients, enhancing statistical power and result reliability. Utilizing a rigorous imaging protocol with specific CT scan parameters ensures consistency, while detailed statistical analyses provide nuanced insights into the spleen size-LI relationship. Ethical considerations are meticulously addressed, including adherence to guidelines and anonymization of patient data. Collaborative interpretation of results minimizes bias, and comprehensive inclusion/exclusion criteria ensure a homogenous study population. Data management protocols prioritize integrity and privacy. These strengths underscore the study's scientific rigor and its potential to inform clinical practice regarding splenomegaly and its implications in COVID-19 prognosis.

Retrospective study designs inherently carry limitations, including potential biases in data collection and reliance on existing records, limiting causal relationship establishment. A single-hospital setting may restrict generalizability due to variations in demographics, protocols, and imaging techniques. Convenience sampling introduces selection bias, impacting representativeness. The absence of a non-COVID-19 splenomegaly control group hinders specificity determination. The sole focus on spleen size and LI, without considering confounding factors, may influence their relationship assessment. Relying solely on spleen size may miss functional aspects of COVID-19. A cross-sectional approach lacks longitudinal data for disease course assessment. Future research should involve prospective, multicenter studies, incorporating control groups and diverse variables, advanced imaging for spleen assessment, and exploring underlying mechanisms of splenic involvement in COVID-19 through international collaborations.

## Conclusions

Our retrospective study explored the correlation between splenomegaly and LI in COVID-19 patients, revealing a marginal positive association between spleen size and the extent of lung damage. Although the Pearson correlation coefficient and simple linear regression analysis indicated a slight linear relationship, the overall impact of spleen size on LI proved minimal, suggesting the presence of other influencing factors not captured in this analysis. The findings highlight the complexity of extrapulmonary manifestations of COVID-19 and highlight the need for further research to fully understand the implications of splenic involvement in the pathophysiology and prognosis of the disease.
